# Hospitalization Rates, Prevalence of Cardiovascular Manifestations and Outcomes Associated With Amyloidosis in the United States

**DOI:** 10.7759/cureus.14177

**Published:** 2021-03-29

**Authors:** Oreoluwa D Oladiran, Adeolu O Oladunjoye, Rashmi Dhital, Olubunmi O Oladunjoye, Ifeanyi Nwosu, Anthony Licata

**Affiliations:** 1 Cardiology, Reading Hospital Tower Health, West Reading, USA; 2 Medical Critical Care, Boston Children's Hospital, Boston, USA; 3 Psychiatry, Reading Hospital Tower Health, West Reading, USA; 4 Internal Medicine, Reading Hospital Tower Health, West Reading, USA; 5 Internal Medicine, Maimonides Medical Center, New York, USA

**Keywords:** amyloidosis, hospitalization, cardiac amyloidosis, takosubo cardiomyopathy, attr amyloidosis, in-hospital outcome

## Abstract

Background

Amyloidosis is a multisystem disease characterized by the deposition of misfolded insoluble precursor protein fibrils in several organs including the heart. Cardiac amyloidosis can result in a wide range of complications that may lead to significant morbidity and mortality. However, contemporary data in the United States (US) on cardiac amyloidosis is scarce despite these negative consequences. In view of this lack of contemporary data, we sort to assess the prevalence, trends of hospitalization, and outcomes of cardiovascular manifestations in amyloidosis. We also explored this retrospective data for factors that may be associated with in-hospital mortality of amyloidosis hospitalization.

Methods

We used the national (nationwide) inpatient sample (NIS) database from January 1, 2007 - December 31, 2014, of adult hospitalizations. We studied the prevalence and trends of hospitalizations of amyloidosis among patient with or without cardiovascular co-morbidities.

Results

We identified 137,797 amyloidosis hospitalizations from 2007 to 2014 of which 87,164 (63.2%) had cardiovascular manifestations. The overall mean age was 70.3±12 years. There were more males (54.5%) overall. The trend of amyloidosis hospitalizations increased significantly from 2007 through 2014 (34 to 73 per 100,000, P_trend_ <0.001) and in-hospital mortality decreased from 8.4 to 6.8 per 100 amyloidosis hospitalizations, P_trend_ <0.001).

Conclusion

Our study showed that hospitalizations of amyloidosis have increased considerably over the past decades with a concurrent decline in in-hospital mortality. Despite this decline and after adjusting for other factors, amyloidosis hospitalization with cardiovascular manifestations was still associated with higher in-hospital mortality. Screening of patients with amyloidosis for cardiovascular manifestations should be more accessible to prevent undesired outcomes.

## Introduction

Amyloidosis is a multisystemic disease that results from misfolding of insoluble precursor protein fibrils that aggregate to cause extracellular disruption of the structural and functional make-up of an organ [1]. This extracellular infiltration of organs leads to disruption of their normal structure and function. It affects several organs including the heart, kidneys, gastrointestinal tract, peripheral nervous system, and soft tissues [2]. Cardiac amyloidosis can result in progressive biventricular wall thickening and stiffness resulting in restrictive cardiomyopathy with concentric ventricular remodeling and low cardiac output [2, 3]. It can present as heart failure with preserved ejection fraction, unexplained left ventricular hypertrophy, or heart failure refractory to therapy. The major types of cardiac amyloidosis are amyloid light (AL) chain amyloidosis and amyloid transthyretin (ATTR) which could either be wild type amyloidosis (ATTRwt) or mutant/hereditary amyloid transthyretin (ATTRm) [2].

AL is the most common and severe type of cardiac amyloidosis with a prevalence of about 70% in newly diagnosed patients [4, 5]. Generally, the epidemiology of AL is poorly reported with as many as eight to 12 per million persons per year in the western countries [5, 6]. In the United States (US), the most comprehensive study conducted was in Olmstead county, Minnesota from 1950 - 1989 by Kyle et al. They reported an incidence rate of nine (CI 5.1-12.8) cases per million persons per year. Also, patients with AL have a poor prognosis with a median survival between six months to three years while ATTRwt has a median survival between three to five years [7, 8].

However, data for ATTR is sparsely reported with about two million inhabitants per year in a certain European country data [9]. Unfortunately, there is little or no availability of recent population-based epidemiological data for cardiac amyloidosis. Therefore, in view of this lack of contemporary data on cardiac amyloidosis, we sort to assess the prevalence and trends of hospitalization and outcomes of amyloidosis with or without cardiovascular comorbidities from 2007 to 2014. We also explored this retrospective data for factors that may be associated with in-hospital mortality of amyloidosis hospitalization.

## Materials and methods

We used the national (nationwide) inpatient sample (NIS) database from January 1, 2007 - December 31, 2014, of adult hospitalizations (≥18 years old). The NIS is a subset of the healthcare cost and utilization project (HCUP) sponsored by the agency of healthcare research and quality (AHRQ). It is the largest all-payer publicly available inpatient care database in the US. The NIS contains data on about 20% sample of all discharges from HCUP participating hospitals and has information on over seven million discharges per year unweighted and 35 million discharges per year weighted [10]. The weighted estimate of which represents >95% of all hospitalized US population. The study database is de-identified and publicly available therefore no ethical clearance or patient consent was sought.

The international classification of diseases ninth revision clinical modification (ICD-9-CM) was used to identify the study population. Patients who had a diagnosis of amyloidosis were identified based on ICD-9 code 277.30 or 277.39 except 277.31 which codes for familial Mediterranean fever. We studied the prevalence and trends of hospitalizations of cardiac amyloidosis patients with or without cardiac manifestations. Cardiovascular manifestations were defined by the presence of coronary artery disease, conduction disorders, arrhythmias, heart failure and non-ischemic cardiomyopathy. We identified these cardiovascular manifestations using ICD-9 CM codes used for billing purposes. These ICD codes were ST-segment elevated myocardial infarction - 410.1, 410.2, 410.3, 410.4, 410.5, 410.6, 410.8, 410.9; non-ST segment elevated myocardial infarction - 410.7; unstable angina - 411.1, 411.8; old myocardial infarction- 412; coronary artery disease - 414.00-414.07; ischemic cardiomyopathy - 414.8; conduction disorder - 426.0, 426.10, 426.11, 426.12, 426.13, 426.2, 426.3, 426.4, 426.5, 426.6, 426.7, 426.8, 426.81, 426.82, and 426.9; arrhythmias - 427.0, 427.1, 427.2, 427.31, 427.32, 427.41, 427.42, 427.5, 427.6, 427.8, and 427.9; heart failure - 428.0, 428.1, 428.2, 428.3, 428.4, and 428.9; pulmonary hypertension - 416.0, 416.8, and 416.9; cardiomyopathies - 425.

Primary outcomes were trends of hospitalization and in-hospital mortality associated with amyloidosis. Additionally, we derived trends of implantable cardioverter defibrillators (ICD), cardiac resynchronization therapy (CRT) and permanent pacemaker (PPM) placement calculated in percentages. We also derived factors associated with in-hospital mortality by using logistic regression model. Univariate and multivariate analysis was done to find the odds ratio (OR) of associations of various factors with in-hospital mortality of amyloidosis. Stata Statistical Software: Release 15 (College Station, TX) was primarily used for all analyses.

## Results

We identified 137,797 amyloidosis hospitalizations from 2007 to 2014. Of these hospitalizations, 87,164 (63.2%) had cardiovascular manifestations while 50,633 (36.8%) had no cardiovascular manifestations.

The baseline characteristics are described in Table 1. The mean age was 71±12 years in those with cardiovascular manifestations which were higher compared to those without cardiovascular manifestations with a mean age of 68 ±13 years (p<0.001). There were more males (54.5%) than females (45.5%) in the overall population, even though this was the reverse in the cohort without cardiovascular manifestations. Amyloidosis hospitalizations were more frequent among whites compared to blacks and other races (67.9% v 20.0% v 12.1%). This also cuts across the two groups, with or without cardiac manifestations. Comorbidities such as diabetes mellitus, hypertension, chronic obstructive pulmonary disease (COPD), hyperthyroidism, dyslipidemia, and obesity were more prevalent in the group with cardiovascular manifestations (Table 1). Most amyloidosis hospitalization overall had medicare insurance (70.6%) compared to other forms of insurance (p<0.001). The mortality prevalence in amyloidosis hospitalization was 7.6% overall, 8.9% in those with cardiovascular manifestations, and 5.4% in those without cardiovascular manifestations (p<0.001).

**Table 1:** Baseline Characteristics of Amyloidosis Hospitalizations Stratified by With or Without Cardiovascular Manifestation n: sample number; N: weighted average estimate; %: percentage

Name	Overall Amyloidosis (n=27,719) (N= 137,796.92)	With Cardiovascular Manifestations (n= 17,531) (N=87,163.78)	Without Cardiovascular Manifestations (n=10,188) (N=50,633.14)	P-Value
Age, years	70.3	71.7	68.0	<0.001
Sex, %				
Male	54.5	58.7	47.2	
Female	45.5	41.3	52.8	<0.001
Race, %				
White	67.9	67.0	69.4	
Black	20.0	22.2	16.0	
Other	12.1	10.8	14.5	<0.001
Comorbidities, %				
Diabetes Mellitus	21.5	22.9	18.9	<0.001
Hypertension	64.5	65.1	64.1	0.06
Fluid and electrolyte disorder	19.3	20.1	17.8	<0.001
Chronic Pulmonary disease	11.5	13.6	7.7	<0.001
Hyperthyroidism	0.4	0.4	0.3	0.31
Dyslipidemia	33.7	34.9	31.7	<0.001
Tobacco	17.4	17.3	17.7	0.50
Obesity	4.6	4.8	4.1	0.0063
Income				
Low-income quartiles	46.4	46.4	46.5	
High-income quartiles	53.6	53.6	53.5	0.92
Insurance				
Medicare	70.6	73.1	66.3	
Medicaid	5.4	4.7	6.8	
Private	20.6	19.3	22.9	
Self-pay	1.5	1.2	1.9	
No charge	0.2	0.2	0.2	
Other	1.7	1.5	1.9	<0.001
Mortality				
No	92.4	91.1	94.6	
Yes	7.6	8.9	5.4	<0.001

The trend of amyloidosis hospitalizations and unadjusted in-hospital mortality

**Figure 1:**
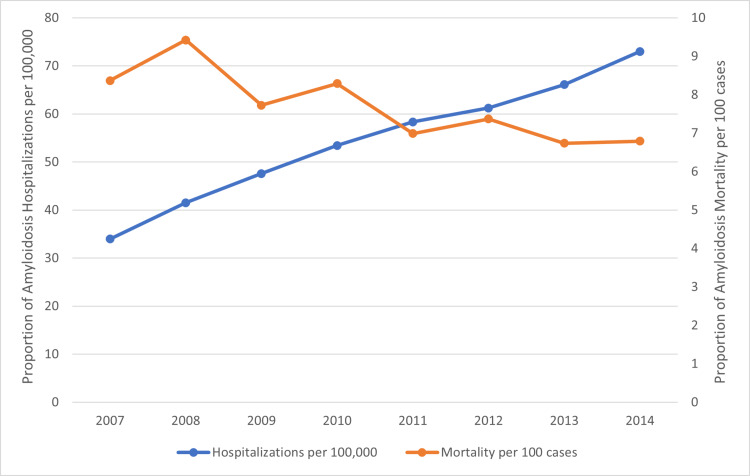
Rate of Hospitalizations and In-Hospital Mortality in Amyloidosis Hospitalizations from 2007 to 2014 The figure shows estimated amyloidosis hospitalizations per 100 000 (orange circle), and unadjusted in-hospital mortality per 100 amyloidosis hospitalizations (blue circle) in the given year

The trend of amyloidosis hospitalizations increased significantly from 2007 through 2014 (34 to 73 per 100,000, Ptrend <0.001) (Figure 1). On the other hand, rates of unadjusted in-hospital mortality decreased from 2007 -2014 (8.4 to 6.8 per 100 amyloidosis hospitalizations, Ptrend <0.001) (Figure 1).

Prevalence of cardiovascular manifestation and rates of ICD, CRT and PPM implantation.

**Figure 2:**
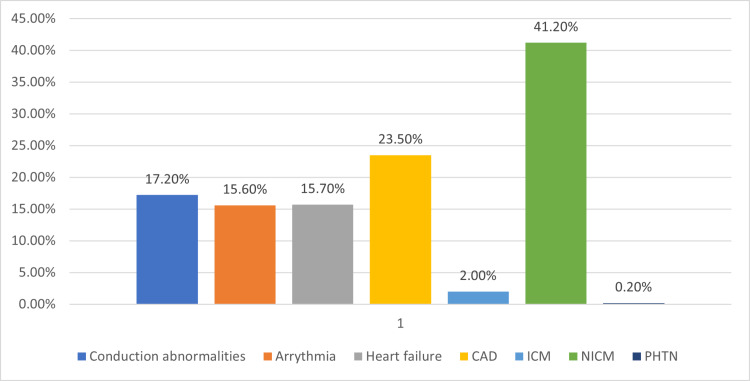
Prevalence of Cardiovascular Manifestations in Amyloidosis in the Percentage Overall, 38% of amyloidosis hospitalizations (n=87,164) had cardiovascular manifestations

The prevalence of cardiovascular manifestations is presented as percentages in Figure 2. A total of 38% (n = 87,164) of amyloidosis hospitalization had co-existing cardiovascular manifestations of one or more types. Non-ischemic cardiomyopathy was the highest at 41.2% followed by coronary artery diseases at 23.5%, conduction abnormalities at 17.2%, heart failure at 15.7%, arrhythmia at 15.6%, ischemic cardiomyopathy at 2.0%, and pulmonary hypertension at 0.2%.

**Figure 3:**
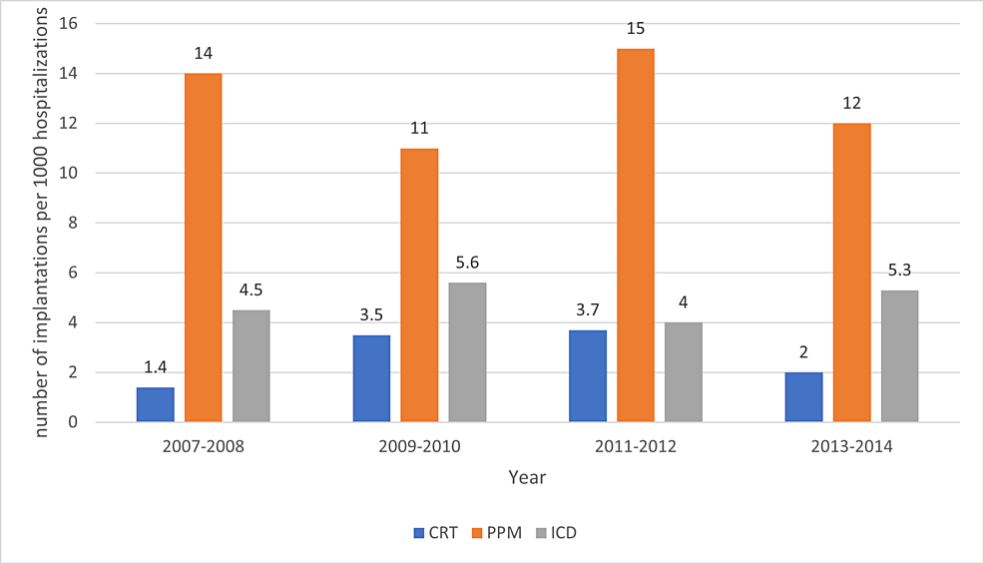
Implantable Cardioverter-Defibrillator, Cardiac Resynchronization Therapy and Permanent Pacemaker Implantations per 1000 Hospitalizations of Amyloidosis from 2007 to 2014 ICD: Implantable cardioverter-defibrillator (ICD), CRT: cardiac resynchronization therapy, PPM: Permanent pacemaker

The rates of CRT and ICD were less than five per 1000 amyloidosis during the study trend across the years. The rates for PPM were steady between 10 -15 per 1000 amyloidosis during the study period (Figure 3).

Association of in-hospital mortality in amyloidosis hospitalization with cardiovascular manifestations

Table 2 shows the factors associated with in-hospital mortality of amyloidosis. After adjusting for age, sex, income, race, and other comorbidities, amyloidosis hospitalization with cardiovascular manifestations was associated with in-hospital mortality adjusted OR of 1.70 (1.51 - 1.92) P<0.001. Excluding ischemic cardiac manifestations, amyloidosis hospitalization with cardiovascular manifestations was still associated with in-hospital mortality adjusted OR of 1.42 (1.28 - 1.57), P<0.001 (Table2).

**Table 2:** Association of In-Hospital Mortality in Amyloidosis With Cardiovascular Manifestations *Odds ratio adjusted for age, sex, income, race, diabetes, hypertension, obesity, tobacco, dyslipidemia, chronic obstructive pulmonary disease, hyperthyroidism, electrolyte disorder. OR: Odds ratio, CI: confidence interval

Name	Unadjusted OR (95% CI), P value	*Adjusted OR (95% CI), P value
Amyloid with cardiovascular manifestations (reference: amyloidosis without cardiovascular manifestations)	1.70 (1.53-1.89), p<0.001	1.70 (1.51 – 1.92), P<0.001
Amyloid with cardiovascular manifestations except for ischemic cardiac disease (reference: amyloidosis without cardiovascular manifestations)	1.46 (1.33 – 1.60), p<0.001	1.42 (1.28 – 1.57), P<0.001

## Discussion

While the hospitalization rate of cardiac amyloidosis in the US has been somewhat documented in the literature, to our knowledge this is one of the few studies that have evaluated the prevalence of cardiovascular manifestations and outcomes associated with amyloidosis in the United States. 

In this study, we report the prevalence of several cardiovascular comorbidities along with the rates of cardiac implantable electronic device (CIED) implantation in this cohort of patients over the study period. Our findings are that about 40% of patients admitted with cardiac amyloidosis had associated non-ischaemic cardiomyopathy.

63% of patients admitted with amyloidosis had some form of cardiovascular disease association. Among those patients, non-ischaemic cardiomyopathy represented the most prevalent cardiovascular disease manifestation, present in about 40% of cases. Amyloidosis hospitalizations progressively increased from 34 per 100,000 admissions in 2007 to about 73 per 100,000 cases in 2014. The mortality rate has however reduced from about 8.4% to 6.8% over the study period. 

Despite the notable increase in hospitalization rate over the study period, there was a steady decline in mortality rate among hospitalized patients. A possible explanation is the increasing awareness and diagnosis of cardiac amyloidosis as well as better management of cardiomyopathies, arrhythmias, and conduction disorders.

Arrhythmia and conduction abnormalities combined represent the second most common cardiovascular disease associated with amyloidosis with a prevalence of 32.8%. However, the rate of CIED implantation has not changed much over the study period, likely due to a lack of data on the effectiveness of device-based therapy in this subset of patients. Cardiac arrhythmias and conduction disorders are common in patients with coronary artery disease (CAD). The amyloid infiltration of the myocardium, conduction tissues, and valves result in arrhythmias and atrial mechanical dysfunction increases the risk of atrial thrombus formation and thromboembolism [11].

CAD as a single entity represents the second commonest cardiovascular manifestations in amyloidosis patients admitted over the study period. It is difficult however to determine if CAD in these patients were a direct consequence of cardiac amyloidosis or multifactorial etiology as a significant population of the study patient had associated CAD risk factors including diabetes, hypertension, dyslipidemia, and smoking history. Coronary amyloidosis may result from the deposition of amyloid fibrils in the coronary vessels leading to CAD, myocardial ischemia, and cardiomyopathy [12]. 

A limitation of this study is the data source which was the NIS database, an administrative database used for billing purposes. It is typically prone to coding errors. The ICD codes used in this study may have been incorrectly coded or in some cases missing. This could have led to underreporting or overreporting cases of cardiac amyloidosis. However, this database has provided the best closest estimate with a large sample size and analytical data with clinical and demographic information from across the US. This potentially increases the power and generalizability of our study findings. 

## Conclusions

Our study showed that hospitalizations of amyloidosis have increased considerably over the past decades, however, the in-hospital mortality is declining. Despite this overall decline in in-hospital mortality, amyloidosis hospitalizations with cardiovascular manifestations showed an increasing trend in hospitalizations and associated with in-hospital mortality, after adjusting for other factors. The presence of cardiovascular diseases in patients with amyloidosis should be considered a strong risk marker for mortality and aggressive measures must be taken to modulate them. 
